# Intake and performance of lambs finished in feedlot with wet brewer’s grains

**DOI:** 10.1186/s40781-018-0166-8

**Published:** 2018-05-14

**Authors:** Mônica Feksa Frasson, Sérgio Carvalho, Gustavo Jaurena, Aliei Maria Menegon, Marcelo Machado Severo, Juliano Henriques da Motta, William Soares Teixeira

**Affiliations:** 1 Department of Animal Science, Federal University of Santa Maria – UFSM, Av. Roraima 1000, Santa Maria, CEP 97105–900 Brasil; 2 School of Agriculture - Department of Animal Science, University of Buenos Aires, Buenos Aires, Argentina

**Keywords:** Feed conversion, Ovines, Weight gain

## Abstract

**Background:**

The use of agroindustrial by-products in ruminant nutrition to be an interesting alternative in order to reduce production costs and environmental impacts arising from the inadequate destination of residues. The initial step of beer production yields a large volume of wet brewer’s grains all year around, which is available at a low cost and has a high nutritional quality, and hence a big potential for animal production.

**Methods:**

Twenty-four Suffolk non-castrated male lambs, from simple parturition were kept in individual spots and allocated to four treatments constituted by four levels of substitution of sorghum silage by WBG (i.e.0; 33.5; 66.5 and 100% of substitution). It was used roughage: concentrate rate of 50:50, based on dry matter.

**Results:**

The ether extract intake increased while the acid detergent fiber intake decreased linearly (*P* ≤ 0.05). Substituion of sorghum silage by WBG did not change lambs’ feed DMI, daily weight gain and feed conversion.

**Conclusion:**

The substitution of sorghum silage by WBG as roughage showed to be a viable alternative from the productive and economic point of view for finishing of feedlot lambs.

## Background

In the last 30 years, the main objectives of sheep production systems have changed from wool to meat production, due to consumer demands accompanied by animal breeding and nutritional improvements. The main meat productive category is lambs, which have high feed conversion, body weight gain and carcass quality, concomitantly with high nutritional requirements during the first months of life [1].

Lamb feedlot fattening systems are an important alternative to stabilize meat quality and product offer throughout the year. Among other benefits to the production systems traditionally used, it may be highlighted the improvement on the animals sanitary condition, money turnover rate, uniformity of meat quality all year around, reduction of slaughter age and the availability of field and forage areas of pasture for the other categories of the herd [2].

Agroindustrial residues are unconventional protein or energetic sources of great potential value for farmers in the effort to intensify and reduce costs, they also reduce the environmental impacts from the inadequate destination of residues [3]. The beer industry yields a large volume of wet brewer’s grains (*WBG*) all year around, which is available at a low cost and has a high nutritional quality, and hence a big potential for animal production. This by-product has a high quality variation; the crude protein concentration varies from 170 to 350 g/kg dry matter –*DM* [4] and Neutral Detergent Fiber (*NDF*) from 550 to 650 g/kg DM [5].

This work was conducted with the objective of assessing the voluntary intake and meat production of intensive reared lambs fed with wet brewer’s grains as roughage feed in substitution of sorghum silage.

## Methods

The work was performed at the Sheep Department of the Polytechnic School of Federal University of Santa Maria (*UFSM*) between October and December 2013. The experiment was approved by the Ethics Committee on Animal Use of the same institution (Protocol N° 037/2014). Laboratory analysis were performed at the Laboratory of Animal Nutrition (Federal University of Santa Maria, Rio Grande do Sul, Brazil).

Twenty-four Suffolk non-castrated male lambs, from single parturition were weaned with an average age of 60 days, properly dewormed (IVOMEC Ovinos 1000 mL – Ivermectina Merial a 0,08%) and inoculated against clostridium (SINTOXAN^®^ Polivalente). After being weaned, animals were allocated to individual stalls (2 m^2^ each) within a shelter, with rice hull bedding, and equipped with individual feeders and drinking fountains. The lambs were allocated in a complete randomized experimental design (*CRD*) with four treatments, constituted by different levels of substitution of roughage (sorghum silage) by WBG (0%; 33%; 66% and 100% of substitution) and six repetitions.

The lambs were weighted and assessed by condition score (scale from 1 to 5; [6]) at the beginning of the experiment, and every 14 days with an 18 h fasting [7]. The experiment started after 10 days of adaptation (facilities, feeding and handling conditions), and extended until animals were slaughtered when body condition score = 3.

The WBG used on the present study was acquired in a brewing agribusiness in Santa Maria and was preserved as silage. The diets were isonitrogenous and formulated according to NRC [8] in order to obtain 200 g of daily weight gain. Animals were fed ad libitum (15% leftovers) offering equal quantities at 08:00 h and 17:00 h. The ration (50:50 roughage: concentrate ratio, on dry matter basis) combined a concentrate (ground corn, soybean meal and mineral mixture) and the roughage, according to the experimental treatment (sorghum silage and WBG; Tables 1 and 2).

**Table 1:** Chemical composition on a dry matter basis (g/kg) of ingredients used in the experimental diets

	Chemical composition (g/kg)						
	Sorghum silage	Wet brewer’s grains	Ground corn	Soybean meal	Calcitic limestone	Dicalcium phosphate	Salt
DM	325	275	884	880	1000	1000	1000
OM	955	913	985	932	–	–	–
CP	42	244	96	531	–	–	–
EE	14	67	44	21	–	–	–
aNDFom	645	560	140	146	–	–	–
ADF	392	225	44	98	–	–	–
TCH	898	602	844	380	–	–	–
NFC	254	42	704	234	–	–	–
ME (Mcal/kg DM)	2,20^1^	2,60^1^	3,08^1^	2,85^2^	–	–	–
Hemic	253	335	–	–	–	–	–
Cell	311	142	–	–	–	–	–
ADL	81	83	–	–	–	–	–
Ashes	45	87	15	68	–	–	1000
Ca	3	2	0,3	3	340	220	–
P	2	7	2	6	0.2	190	–

*DM* dry matter, *OM* organic matter, *CP* crude protein, *EE* ether extract, *aNDFom* neutral detergent fiber, *ADF* acid detergent fiber, *TCH* total carbohydrates, *NFC* non-fibrous carbohydrates, *ME* metabolizable energy, *Hemic* hemicellulose, *Cell* cellulose, *ADL* lignin, ashes, *Ca* calcium, *P* phosphorus

^1^Estimated by National Forage Testing Association [14].^2^Estimated by Van Soest [15]

**Table 2:** Proportion of ingredients (% DM) and chemical composition (g/Kg) of dry matter in the experimental diets.

	Wet brewer’s grains levels (%)			
	0	33	66	100
Proportion of ingredients (% DM)				
Fiber				
Sorghum silage	50.0	33.2	16.7	0.0
Wet brewer’s grains	0.0	16.7	33.2	50.0
Concentrate				
Ground corn	18.4	26.2	33.9	41.6
Soybean meal	28.1	20.3	12.6	4.8
Calcitic limestone	1.5	1.8	2.1	2.4
Dicalcium phosphate	1.0	0.6	0.3	0.0
Salt	1.0	1.0	1.0	1.0
Total	100	100	100	100
Chemical composition (g kg^−1^ DM)				
Dry matter level	607	600	591	583
Organic matter	920	920	915	912
Crude protein	188	188	188	188
Ether extract	21	32	42	53
Neutral detergent fiber	389	374	360	345
Acid detergent fiber	232	200	170	136
Total carbohydrates	714	700	685	671
Non-fibrous carbohydrates	322	323	325	326
ME (Mcal/kg dry matter)^1^	2,47	2,55	2,64	2,72
Ashes	80	82	84	89
Calcium	10	10	10	10
Phosphorus	5	5	5	5

^1^Metabolizable energy

Every three days, feeds and leftovers samples were collected and every 21 days a pool sample was composed, stored in plastic bags at − 18 °C for subsequent laboratory analysis. For chemical analysis, ingredients samples and leftovers were dried at 55 °C for 72 h. Subsequently, samples were ground (2-mm screen; Willey type mill, Arthur H. Thomas, Philadelphia, PA) and analyzed for dry matter (*DM*; Method 934.01 [9]), organic matter (*OM*; Method 942.05 [9]), ash (Method 942.05), and ether extract (*EE*; Method 920.39 [9]). The Crude Protein content (*CP* = N × 6.25) was determined by Kjeldahl (Method 984.13 [9]).

Neutral Detergent Fiber was expressed free of residual ash (*aNDFom***;** Van Soest et al. [10]), and using heat-stable α-amylase and without sulphite (based on Mertens [11]). Acid detergent fiber (*ADF*) was assessed according to Method 973.18 of AOAC [12]; both method were done by using an autoclave at 110 °C for 40 min [13]. For Acid detergent lignin (*ADL*), bags containing residual ADF were treated with 12 M H_2_SO_4_ for 3 h (Method 973.18; [12]).

Non-fibrous carbohydrates (*NFC*; % in the DM) were computed as 100 – (CP + NDF + EE + ash) [11], and Total carbohydrates (*TCH*) as 100 - (%CP + %EE + % ASH). Metabolizable energy (*ME, Mcal kg*^*-1*^) was computed as ME = %TDN/(4.4 × 0.82), while %TDN = 31.4 + (53.1 x NEL) for silage [14] and ME (Mcal/kg DM) = 3,32–0,055 x %ADF for corn grain [14], ME (Mcal/kg DM) = 3,6/100 × ((100-%FDN) × 0,98 + %FDN×(1473–0,789 log10 (LDA/FDA × 100))–12,9) for soybean meal [15].

Voluntary feed intake was computed through the difference between offered and leftovers (on DM basis) for DM, OM, CP, EE, NDF, ADF, TC, NFC and TDN.

Results were analyzed according to a completely randomized experimental design with four experimental diets, and six repetitions. Analysis of Variance and Regression Analysis were performed using SAS statistical package [16]. Differences among treatment means were declared whenever *P* ≤ 0.05.

## Results and discussion

The WBG by-product had higher concentrations of CP and EE than Sorghum silage, but aNDFom, ADF and ADL concentrations were similar for both feeds (Table 1). The chemical characteristics of WBG were coherent with typical values reported by Feedipedia [17], though ashes and LDA were higher. On the other hand, sorghum silage composition was typical for Brazil and very similar to values tabulated by Valadares Filho et al. [18]. The diets composition reflected the different sorghum silage/WBG ratios, with increasing values of EE and ME and decreasing values of aNDFom and ADF as the proportion of WBG increased.

When sorghum silage was used as the unique supplement, ADF concentration (232 g kg^− 1^ DM) was higher than that observed for 100% WBG inclusion (136 g kg^− 1^ DM; Table 2); the rest of the treatments had ADF concentrations in accordance with sorghum silage: WBG ratio. Normally, higher ADF values would reduce the performance of lambs as a consequence of the expected reduction in digestibility (Van Soest, [15]).

In this study, voluntary feed intake was higher (4.12% body weight (*BW*); Table 3) than predicted by NRC [8] for late maturity lambs (i.e. 2.97% of BW) and was not influenced (*P* > 0.05) by WBG proportion in the final diet (Table 2). This was a probable consequence of the similar NDF concentration in all the experimental diets, which lead to a range of NDF intakes from 1.24 to 1.50% BW. Lambs daily intakes were higher than those observed by Kozloski et al. [19], who found that sorghum silage could be incorporated into the ration up to *c.a.* 30% of DMI (2.7% BW) or *c.a.* 0.80% BW of NDF intake. Lack of differences were also reported in dairy cows by Geron et al. [20] (inclusion up to 15% of fermented brewer’s residue in diets containing also corn silage, pre-dried ryegrass silage and concentrate), and other authors using WBG up to 16% [21] and 10% on a DM basis [22]. As DMI was not affected by treatments, changes in NDF consumption as % BW reflected changes in NDF ration concentration, hence that the high levels of daily DMI recorded (4.03% BW) and the trend to reduce DMI as WBG increased would lead to speculate that animals were within the chemostatic intake regulation range.

**Table 3:** Average daily intake according for the experimental rations

	Percentage of wet brewer’s grains						
	0	33	66	100	Regression equation	R^2^	Pr > F
Intake as % of body weight (Mean ± Standard deviation)							
DM	4.04 ± 0.5	4.26 ± 0.4	3.98 ± 0.5	3.83 ± 0.4	Ῡ = 4.03	–	0.29
OM	3.73 ± 0.4	3.92 ± 0.3	3.67 ± 0.5	3.53 ± 0.4	Ῡ = 3.71	–	0.28
CP	0.81 ± 0.1	0.83 ± 0.1	0.76 ± 0.1	0.70 ± 0.1	Ῡ = 0.77	–	0.02
EE	0.08 ± 0.01	0.13 ± 0.01	0.16 ± 0.02	0.19 ± 0.02	^1^	0.83	<.0001
NDF	1.44 ± 0.2	1.50 ± 0.2	1.33 ± 0.2	1.24 ± 0.2	Ῡ = 1.37	–	0.03
ADF	0.84 ± 0.1	0.77 ± 0.1	0.60 ± 0.1	0.45 ± 0.1	^2^	0.73	<.0001
TCH	2.84 ± 0.3	2.95 ± 0.2	2.74 ± 0.3	2.63 ± 0.3	Ῡ = 2.80	–	0.17
NFC	1.38 ± 0.2	1.43 ± 0.1	1.38 ± 0.1	1.36 ± 0.2	Ῡ = 1.38	–	0.68
Intake as kg/day in DM (Mean ± Standard deviation)							
DM	1.30 ± 0.2	1.44 ± 0.2	1.38 ± 0.3	1.26 ± 0.2	Ῡ = 1.35	–	0.68
OM	1.20 ± 0.2	1.33 ± 0.2	1.27 ± 0.2	1.16 ± 0.2	Ῡ = 1.24	–	0.66
CP	0.26 ± 0.04	0.28 ± 0.05	0.26 ± 0.05	0.23 ± 0.04	Ῡ = 0.26	–	0.18
EE	0.02 ± 0.005	0.04 ± 0.01	0.06 ± 0.01	0.06 ± 0.01	^3^	0.63	<.0001
NDF	0.46 ± 0.1	0.50 ± 0.1	0.46 ± 0.1	0.41 ± 0.1	Ῡ = 0.46	–	0.23
ADF	0.27 ± 0.04	0.26 ± 0.05	0.20 ± 0.05	0.15 ± 0.03	^4^	0.53	<.0001
TCH	0.91 ± 0.1	1.00 ± 0.1	0.95 ± 0.2	0.87 ± 0.1	Ῡ = 0.93	–	0.53
NFC	0.44 ± 0.1	0.49 ± 0.1	0.48 ± 0.1	0.45 ± 0.1	Ῡ = 0.46	–	0.98
ME Mcal/day	3.26 ± 0.5	3.72 ± 0.6	3.68 ± 0.7	3.51 ± 0.5	Ῡ = 3.54	–	0.52

*DM* dry matter, *OM* organic matter, *CP* crude protein, *EE* ether extract, *NDF* neutral detergent fiber, *ADF* acid detergent fiber, *TCH* total carbohydrates, *NFC* non-fibrous carbohydrates, *ME* Metabolizable energy

^1^- Ŷ = 0.08276 + 0.00121× WBR

^2^- Ŷ = 0.86761–0.00375× WBR

^3^- Ŷ = 0.02698 + 0.00041047× WBR

^4^- Ŷ = 0.28136–0.00114 × WBR

Similarly, differences in chemical fractions intake were accounted for changes in feedstuffs composition. Hence that as WBG proportion increased EE intake was raised (*P* < 0.001; Table 3), reaching a maximum of 5% of total DMI when 100% of WGB was used as supplement. According to Willians [23], this supply should be limited to a maximum of 5% to avoid negative effects on dietary fiber digestibility and DM intake. Consequently, it may be concluded that EE did not reach levels that could reduced intake.

Growth rate, feed conversion and body condition score did not change with rate of WBG inclusion (Table 4) even when diets with higher WBG levels had higher concentrations of ME and lower concentrations of ADF (Table 2). However, the impact of higher WBG inclusion it is also expected to have increased by-pass protein supply as suggested by other authors reports Clark et al. [24] and Satter [25].

**Table 4:** Average values of initial body weight (IBW), final body weight (FBW), daily weight gain (DWG), feed conversion (FC) and body condition score (BCS) according to the sorghum silage replacement levels by wet brewer’s grains

	Diets						
	0	33	66	100	Regression equation^1^	R^2^	Pr > F
Performance of lambs (Mean ± Standard deviation)							
IBW (kg)	24.7 ± 4.0	25.8 ± 5.2	25.0 ± 3.8	25.6 ± 4.0	Ῡ = 25.3	–	0.78
FBW (kg)	39.3 ± 5.3	40.5 ± 3.3	42.2 ± 5.0	39.0 ± 5.3	Ῡ = 40.2	–	0.95
DWG (kg d^−1^)	0.25 ± 0.1	0.33 ± 0.1	0.37 ± 0.1	0.32 ± 0.1	Ῡ = 0.32	–	0.21
FC	5.1 ± 0.3	4.5 ± 0.8	3.9 ± 0.8	4.4 ± 1.0	Ῡ = 4.5	–	0.15
BCS^1^	3.2 ± 0.3	3.0 ± 0.3	3.1 ± 0.2	3.1 ± 0.3	Ῡ = 3.1	–	0.70

^1^Body condition score: 1 = very poor – 5 = very fat

The positive response to WBG recorded in our experiment was *c.a*. twofold that observed by McCarthy et al. [26], who worked similar lambs, but this difference was probably due to the higher energy content of control treatment in their experiment. In other experiment an improvement on the body weight gain was resported until a 50% substitution rate of lucerne hay by dried brewers grains [27].

## Conclusion

The main objective of this work was to assess the voluntary intake and animal performance of intensive reared lambs fed with WBG in replacement of sorghum silage.

The WBG had higher concentrations of CP and EE than Sorghum silage, and its chemical composition was typical of these brazilian by-products. Substituion of sorghum silage by WBG did not change lambs’ feed DMI, DWG and FC.

## Abbreviations

ADF: Acid detergent fiber
ADL: Acid detergent lignin
aNDFom: Neutral Detergent Fiber assayed with a heat stable amylase and expressed exclusive of residual ash
BW: Body weight
CP: Crude Protein
CRD: Complete randomized experimental design
DM: Dry matter
DWG: Daily weight gain
EE: Ether extract
FC: Feed conversion
ME: Metabolizable energy
NDF: Neutral Detergent Fiber
NEL: Net energy for lactation
NFC: Non-fibrous carbohydrates
NRC: National Research Council
OM: Organic matter
TCH: Total carbohydrates
TDN: Total Digestible Nutrients
UFSM: Federal University of Santa Maria
WBG: Wet brewer’s grains
WBG-C: Wet brewer’s grains-ground corn mixture
